# A machine learning analysis of COVID-19 mental health data

**DOI:** 10.1038/s41598-022-19314-1

**Published:** 2022-09-02

**Authors:** Mostafa Rezapour, Lucas Hansen

**Affiliations:** Department of Mathematics, Wake Forest University, Winston-Salem, NC USA

**Keywords:** Psychology, Diseases, Mathematics and computing

## Abstract

In late December 2019, the novel coronavirus (Sars-Cov-2) and the resulting disease COVID-19 were first identified in Wuhan China. The disease slipped through containment measures, with the first known case in the United States being identified on January 20th, 2020. In this paper, we utilize survey data from the Inter-university Consortium for Political and Social Research and apply several statistical and machine learning models and techniques such as Decision Trees, Multinomial Logistic Regression, Naive Bayes, k-Nearest Neighbors, Support Vector Machines, Neural Networks, Random Forests, Gradient Tree Boosting, XGBoost, CatBoost, LightGBM, Synthetic Minority Oversampling, and Chi-Squared Test to analyze the impacts the COVID-19 pandemic has had on the mental health of frontline workers in the United States. Through the interpretation of the many models applied to the mental health survey data, we have concluded that the most important factor in predicting the mental health decline of a frontline worker is the healthcare role the individual is in (Nurse, Emergency Room Staff, Surgeon, etc.), followed by the amount of sleep the individual has had in the last week, the amount of COVID-19 related news an individual has consumed on average in a day, the age of the worker, and the usage of alcohol and cannabis.

## Introduction

In late December 2019, the novel coronavirus (Sars-Cov-2) and the resulting disease COVID-19 were first identified in Wuhan China^1^. The disease slipped through containment measures^2^, with the first known case in the United States being identified on January 20th, 2020^3^. As countries around the world grappled with the new disease, new data and its analysis have heavily influenced policymakers around the globe and vastly transformed our knowledge of the disease and its effects^4^. In this paper, we examine the indirect effects of COVID-19 on the mental health of frontline workers in the United States and offer approaches to help frontline workers retain their mental health during the COVID-19 pandemic.

The United States Center for Disease Control and Prevention (CDC) defines an epidemic as *an increase, often sudden, in the number of cases of a disease above what is normally expected in the population in an area*. The CDC then defines a pandemic as *an epidemic that has spread over several countries or continents, usually affecting a large number of people*^5^. Following these guidelines, COVID-19 was officially declared a pandemic on March 11th, 2020 by the World Health Organization (WHO) and is still considered to be an ongoing pandemic. Prior to COVID-19, the most recent public health crisis was Zika Virus, first isolated in Uganda in 1947^6^. Other recent major infectious disease events include outbreaks of Ebola in 2014, MERS in 2013, SARS in 2003, and H1N1 Influenza (Swine Flu) in 2009.

As the world is battling the COVID-19 pandemic, one of the most vulnerable groups for mental health problems are frontline workers such as nurses, doctors, and emergency room staff. The risks of being on the front lines of combating the COVID-19 pandemic are not well understood^7^ with even less being known about how to ensure the workers remain mentally healthy. Post-SARS research suggests hospital administration and staff needs to recognize that the impact to the health of frontline workers and frequent changing of infectious disease policy can play major roles in detrimentally impacting frontline workers mental health^8^. It is known that survivors of infectious diseases have higher rates of Post-Traumatic Stress Disorder (PTSD)^9^. It is also known that negative outcomes such as anxiety, burnout, and depression have been reported after outbreaks, suggesting possible long-term effects of being on the front-line during health crisis^10^. Moreover, new research has indicated an increase in physician suicide rates during the COVID-19 pandemic^11^. Like physician suicides, during COVID-19 there has been an increase in the in the symptoms of those suffering from psychiatric disorders^12^. Notably in the Republic of Ireland, a study found that during the initial phase of the COVID-19 pandemic, depression became more common^13^.

In this paper, we utilize survey data obtained from the University of Michigan’s Inter-university Consortium for Political and Social Research (ICPSR). The data was collected by Deirdre Conroy^14,15^ from the University of Michigan Department of Psychiatry and Cathy Goldstein from University of Michigan Department of Neurology. According to the ICPSR, “The rationale for this study was to examine whether sleep, mood, and health related behaviors might differ between healthcare workers who transitioned to conducting care from home and those who continued to report in-person to their respective hospitals or healthcare facilities.” The original data contained 916 survey responses. The average survey response answered 94.5% of the survey questions, totaling 29 questions in total, with many questions leaving room for respondents to write how their mood or habits had changed since COVID-19 protocols were in effect. The data was stripped of any identifying information about the respondents and contained both categorical and numeric columns^16^.

In this study, to treat the missing values for the categorical variables appropriately and keep the results more reliable, we have not used machine learning techniques, e.g. imputation. Instead, we have removed the datapoints (rows) with many missing values from the dataset, and as the result, we have ended up with 518 data points. Since the mental health dataset contains categorical and ordinal variables, we have first encoded them to numbers, and then we have used several encoding techniques such as one-hot encoding or dummy variable encoding as well as several packages in Python such as *OneHotEncoder*, *LabelEncoder* or *OrdinalEncoder* from *sklearn.preprocessing* to prepare the dataset for analysis (see Supplementary file for further details about the dataset).

**Figure 1:**
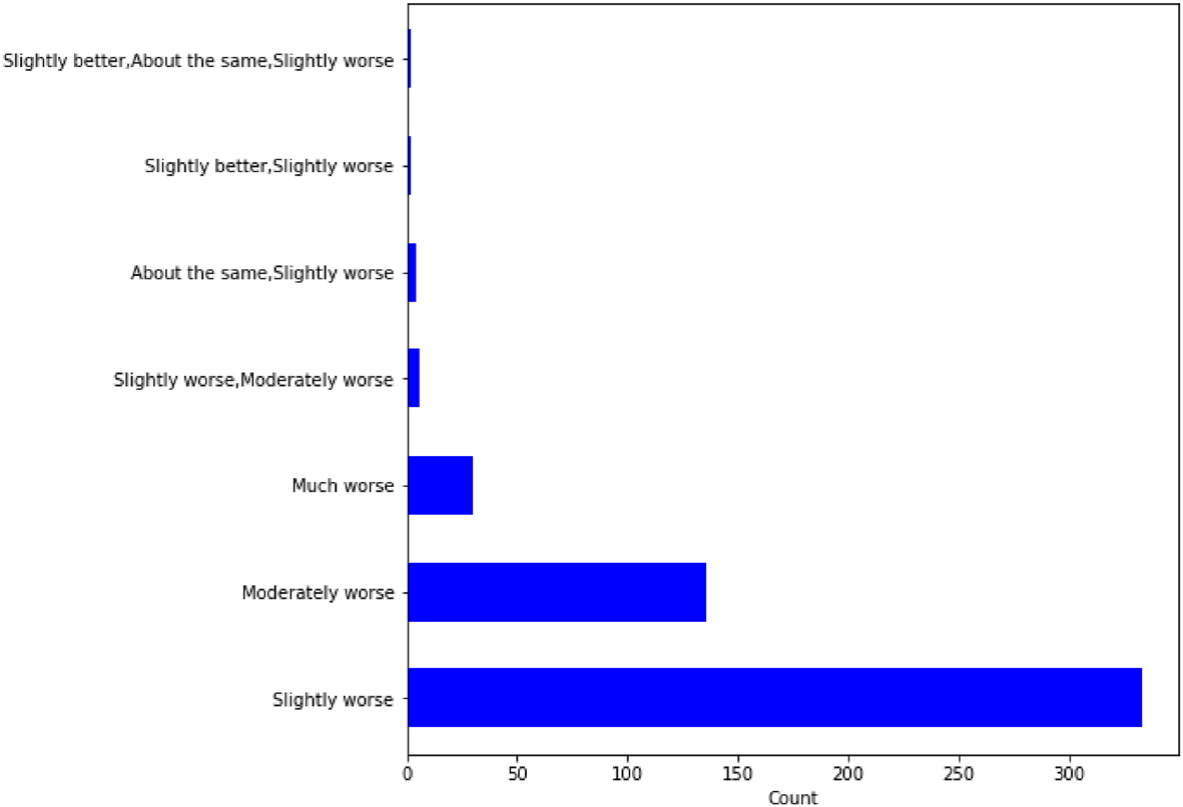
Q29 (a): “Please tell us how your mood has changed. My mood has been:”.

In this paper, Question 29 (a) in the survey, which reads “Please tell us how your mood has changed?” (see Fig. 1) is selected as the target variable. Our goal is to identify the top predictors of mental health decline by examining multiple machine learning models utilizing their feature importance.

The remainder of this paper is structured as follows: “Methods” section discusses the methodology, describes the experimental framework used to find the top predictors of mental health decline among frontline workers in the United States. In “Discussion” section discusses and analyzes the top predictors of mental health decline obtained by Machine Learning methods. Finally, “Conclusion and future work” section concludes the paper by summarizing our overall findings and suggests a direction for future research.

## Methods

The data utilized in this study was taken from the University of Michigan’s Inter-University Consortium for Political and Social Research, and collected by Deirdre Conroy^14^. Conroy et al.^15^ confirms that all experiments were performed in accordance with relevant guidelines and regulations (see the Methods section in^15^). The data is from a survey conducted within the University of Michigan Medical Center^15^. All survey data was collected in accordance with relevant guidelines and regulations. The survey was undertaken after clearing University of Michigan Institutional Review Board (HUM00180147) approval, at which point the Qualtrics survey link was sent via email listservs that would reach large numbers of health care providers^15^. It is noted that no compensation for participation was provided^15^. Additionally, all participants have provided full and informed consent by completing the survey^15^.

### Computational process

Our main goal in this paper is to use multiple supervised and unsupervised machine learning models and techniques to find the top predictors (features) of mental health decline. Only for supervised machine learning models with high accuracy (at least 90%), which are more reliable, we calculate the feature importance scores and proceed feature selection phase to find the top predictors for the mental health decline among frontline workers. Feature importance can be interpreted as the features that were the most valuable for generating the final prediction. Phrased differently, a high feature importance means the feature contains a lot of predictive power.

In this section, we provide an overview and results for all supervised and unsupervised machine learning methods that we use in this paper. To recall, the goal of supervised learning is to predict accurately the value or the class of an unseen data point. To achieve this goal, we have trained a model on a training data and evaluated its accuracy on a test data. In all supervised methods, we have split the data set into two parts, one part (for instance 75% of all observations) as the training data set, and the other part (for instance 25% of all data set) as the test data set. Then, we have trained each model only on the training data set and finally tested the model on the test data set. Each supervised predictor contains some hyper-parameters that control overfitting or underfitting^17^. For instance, hyper-parameters for decision trees, k-nearest neighbors, neural networks are the depth of the tree, *k*, and the number of hidden layers, respectively. There are different techniques, such as the elbow rule, for finding the best value for the hyper-parameters^18^ that have been used in this study. Now, we overview the most common unsupervised and supervised techniques.

#### Unsupervised feature selection

**Figure 2:**
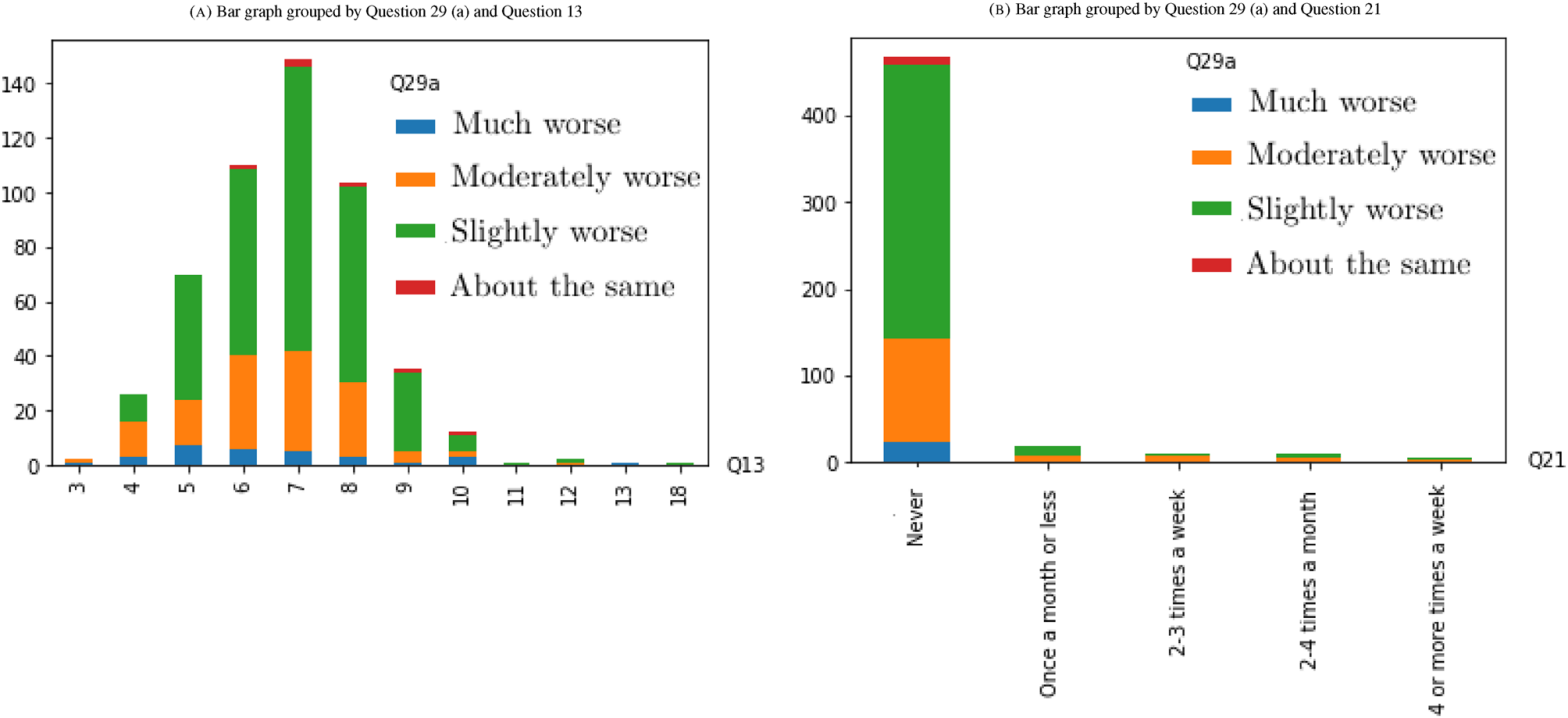
Bar graph grouped for the variables that pass the Chi-Squared test with significance level $\alpha =0.05$.

**Figure 3:**
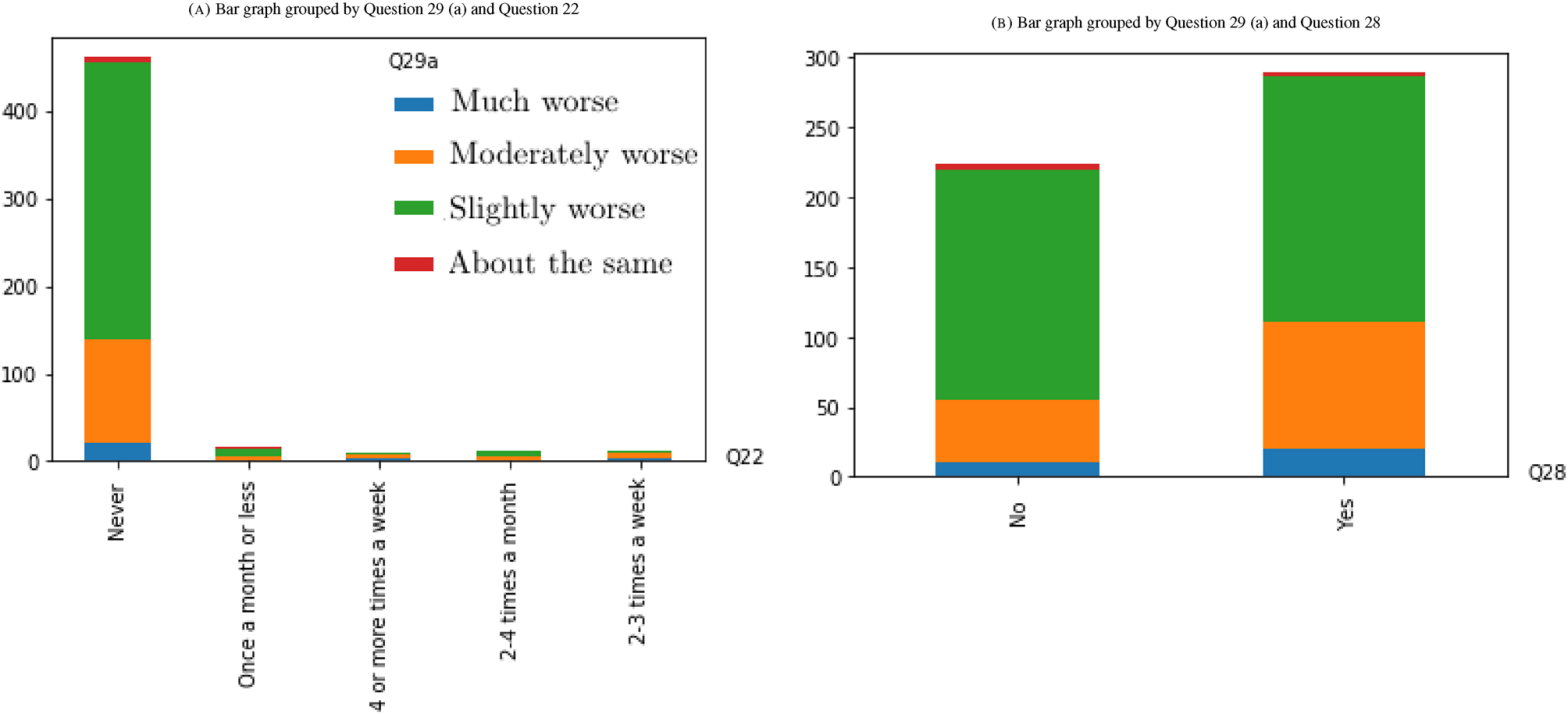
Bar graph grouped for the variables that pass the Chi-Squared test with significance level $\alpha =0.05$.

Since the problem is a classification problem, where the majority of variables are categorical, we can use statistical tests such as Chi-Squared tests to determine whether the target variable, Question 29 (a), is dependent or independent of the rest of variables. The variables that are independent can be considered as candidates for irrelevant features to the problem and they might be removed. During the data preparation process, we collapsed continuous variables into smaller groups (categories) to prepare the dataset for applying a Chi-Squared test^19^. After the data preparation process, we have ended up with a sample size of 513, which vastly exceeds the minimum of 20 to 50 recommended by Rana and Singhal^20^ for avoiding Type II bias (failing to reject the null hypothesis when it is truly false). We first constructed a contingency table, then calculated the expected frequencies for pairs of target variables and each input variable, and finally, applied a Chi-Squared test with significance level $\alpha =0.05$ to determine whether there exists a correlation between frontline workers mental health decline and the rest of factors (features). It turns out that the Chi-Squared test rejects the null hypotheses, $H_0$: Question 29 (a) is independent of Question *i* versus the alternative $H_1$: Question 29 (a) is not independent of Question *i*, for $i \in \{13, 21, 22, 28\}$ (see Table 1, and Figs. 2, 3).

**Table 1:** Chi-Squared test with significance level $\alpha =0.05$ rejects the null hypotheses, $H_0$: Question 29 (a) is independent of Question *i* versus the alternative $H_1$: Question 29 (a) is not independent of Question *i*, for $i \in \{13, 21, 22, 28\}$.

Chi-Squared test rejects the null hypothesis that the target variable is independent of the follwoing variables	
Q13. Approximately how many hours did you sleep on an average work night in the last week?	
Q21. In January 2020, approximately how often did you use marijuana/cannabis (recreational or medical)?	
Q22. In the last month, approximately how often did you use marijuana/cannabis (recreational or medical)?	
Q28. Has the amount of food you have been eating per day changed?	

#### Supervised feature selection

In this subsection, we provide an overview and results for several supervised machine learning methods.

*Decision trees* Decision trees are one of the most popular machine learning algorithms due to visualization simplicity. In other words, when we construct a tree, we can figure out what feature is most relevant at the first glimpse. A decision tree breaks down a dataset into smaller and smaller subsets containing data points that are more homogenous. Given a dataset with many features, different kind of decision trees with different depth can be constructed for training the model. However, finding the optimal decision tree, which is the shortest tree that can predict the class label for any unseen data, is computationally expensive. A decision tree contains a root node, internal nodes and leaf or terminal nodes. In a decision tree classifier, each leaf is assigned for a class label. Moreover, the root node and internal nodes contain feature test conditions. The order of non-terminal roots plays a significant role to find the optimal tree. A decision tree can also be used for feature selection because it works based on the relevance of each feature. The goal of a decision tree is to minimize the entropy of the current sample to the next subsets by splitting the sample appropriately. The entropy for a sample *S* with *n* classes is defined as $E(S)=-\sum _{i=1}^{n}p_ilog({p_i})$, where $p_i$ is the probability of the class *i* in the sample *S*^21^. A simple decision tree model has been utilized on the Mental Health Data, and its accuracy turns out to be 74.12%, which prevents us from analyzing the top predictors of the model.

*Multinomial logistic regression* Logistic regression is used to model binary categorical outcomes because it is improper to use linear regression while the output is not numeric, and the error terms are not normally distributed. In linear regression, parameters are estimated by ordinary least squares (OLS), where the sum of squared deviations of the predicted values from observed values is minimized. However, OLS is not appropriate for logistic regression to find unbiased estimators with minimum variance. For logistic regression, maximum likelihood estimation (MLE) is widely used to estimate parameters. Multinomial logistic regression is an appropriate extension of binary logistic regression for multiclass classification problems. It uses maximum likelihood estimation (MLE) to evaluate the probability of a target variable’s classes^22^. We have used a multinomial logistic regression model on the Mental Health Data, and its accuracy turns out to be 64.71%, which is not satisfactory and prevents us from analyzing the top predictors of the model.

*Recursive Feature Elimination* The Recursive Feature Elimination (RFE) is a machine learning technique for finding the most relevant feature. It works by recursively removing attributes and building a model on those attributes that remain. If the accuracy of a model drops significantly in absence of a feature, it indicates that the feature is important. We have applied the recursive feature elimination using multinomial logistic regression and decision tree in presence and absence of all features. Then we have ranked features according to the reduction of accuracy of a model in absence of a feature at a time. Table 2 displays the top predictors obtained by RFE using simple decision tree and multinomial logistic regression models.

**Table 2:** Applying RFE on a Decision Tree and a Multinomial Logistic Regression.

RFE feature selection using Decision Tree	
Rank	Feature
#1	Q21: In January 2020, approximately how often did you use marijuana/cannabis?
#2	Q24: How many hours of COVID-19 related news or social media are you consuming on average per day?
#3	Q20: In the last month, approximately how often did you have a drink containing alcohol?
#4	Q23: Has the amount of news you are consuming increased since the end of Feb, 2020?

RFE feature selection using Multinomial Logistic Regression	
Rank	Feature
#1	Q18: Has the amount of alcohol you are consuming changed?
#2	Q14: Has the number of your worked hours per week changed?
#3	Q16: Have your sleep patterns changed?
#4	Q20: In the last month, approximately how often did you have a drink containing alcohol?

*Naive Bayes* The Naive Bayes Classifier works based on the Bayesian theorem and is efficient when the dimensionality of the inputs is high. Given a data set comprising *N* observations with their corresponding target class labels and *D* features $X = (x_1,x_2,...,x_d)$, we want to find the posterior probability for the outcome of the event $\Omega$ among a set of possible outcomes $\{y_1,y_2,...,y_K$}. The Naive Bayes classifier is assumed to be applied for datasets that the conditional probabilities of the features are statistically independent,

1 $$\begin{aligned} p(\Omega = y_j|x_1,x_2,...,x_d) \propto p(x_1,x_2,...,x_d|y_j)p(y_j) \Longrightarrow&p(\Omega = y_j|x_1,x_2,...,x_d) \propto p(x_1|y_j)p(x_2|y_j)...p(x_d|y_j)p(y_j). \end{aligned}$$

The Naive Bayes classifier reduces a high-dimensional task to a one-dimensional kernel density estimation. It is an effective and commonly used probabilistic machine learning classifier. Naive Bayes classifiers are especially used for text classification and spam detection^23^. There is very little training in Naive Bayes compared to other common classification methods. We have also used a Naive Bayes classifier, but its accuracy on the Mental Health Data turns out to be 43.52%, which is significantly low.

*k-Nearest neighbors* The k-nearest neighbors algorithm (KNN) classifies a new observation by a majority vote of its neighbors and it does not have the training phase. It calculates the distance from the new observation point to all seen data points, and then the new observation class label is assigned to be the class that is the most common among its *k* nearest neighbors. The KNN should be one of the first choices for a classification when there is little or no prior knowledge about the data and feature labels. The KNN is computationally expensive, especially when the dimension of feature vector is high, because it must store all data points and their distances from the new observation^24^. We have used the KKN model on the Mental Health Data, and its accuracy turns out to be 71.76%, which is not satisfactory.

*Support vector machines* The support vector machine algorithms seek a hyperplane in an *n*-dimensional space (*n* is the number of features) that classifies the data points with the maximum margin. The data points that are closer to the hyperplane and play an important role in finding the best position and orientation of the hyperplane to maximize the margin are called vector machines. One-vs-one Support Vector Machines (SVM OVO) is an appropriate extension of binary for multiclass classification problems. It splits the dataset into one dataset for each class versus every other class, which means it converts a multiclass classification dataset into multiple binary classification problems^25^. We have applied the SVM OVO model on the Mental Health Data, and its accuracy turns out to be 70%, which is not satisfactory.

*Neural networks* Neural networks are a set of algorithms that are designed to recognize patterns. We can consider them as clustering and classification layers on top of the data we store. They predict the label of unseen data according to similarities among the example inputs. Neural networks can also be used for extracting features that are fed to other algorithms for classification. Deep learning is the name that is used for stacked neural networks, which contains several layers. Each layer in the network is made of different neurons, where computations happen to decide whether a neuron should fire, and the signal should progress further through the network. A neuron fires when it meets a sufficient stimulus. A neuron combines the last layer neurons output with a set of weights. This weighted sum is the input of a function called activation function to determine whether and to what extent the signal should progress to affect the ultimate outcome^26^. We have utilized a neural network with 10 hidden layers and SoftMax as the last layer activation function on the Mental Health Data, and its accuracy turns out to be 82.35%. Hyperparameter tuning has also been done, but no better accuracy obtained.

**Figure 4:**
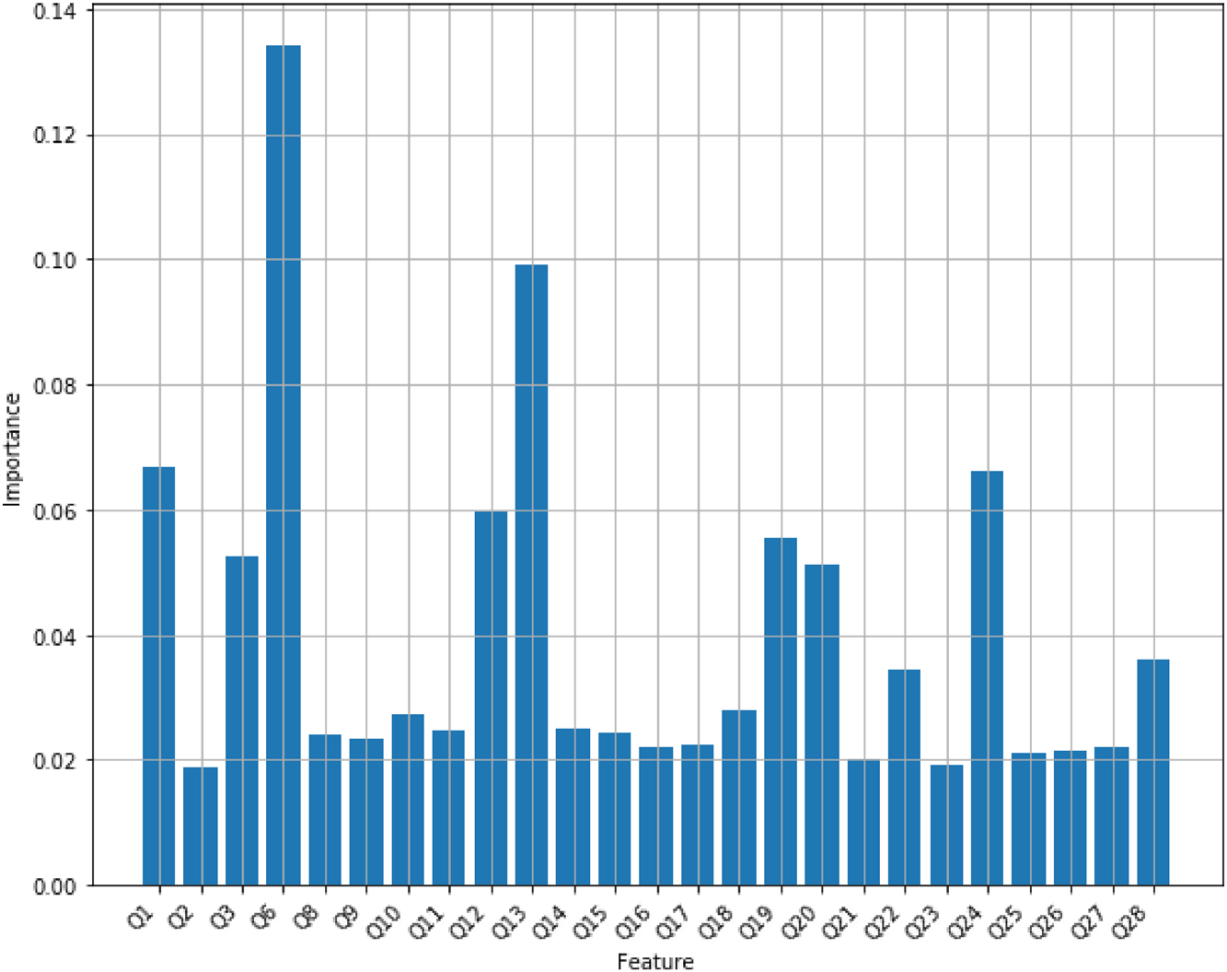
Feature importance scores of Random Forest with 10 trees with maximum depth of 10.

*Random forest* Random Forest Classifier is an ensemble algorithm, which is a model that combines more than one algorithm of same or different kind for classifying objects. From randomly selected subset of training data, random forest generates a set of decision trees, and then it specifies the class label of a data point by aggregating the votes from different decision trees. The random forest is more powerful than a single decision tree classifier because it avoids overfitting on the training data. We have used a regular random forest with 10 trees with maximum depth of 10 on the Mental Health Data, and its accuracy turns out to be 80.59%. Figure 4 displays feature scores of the random forest model with 10 trees.

**Figure 5:**
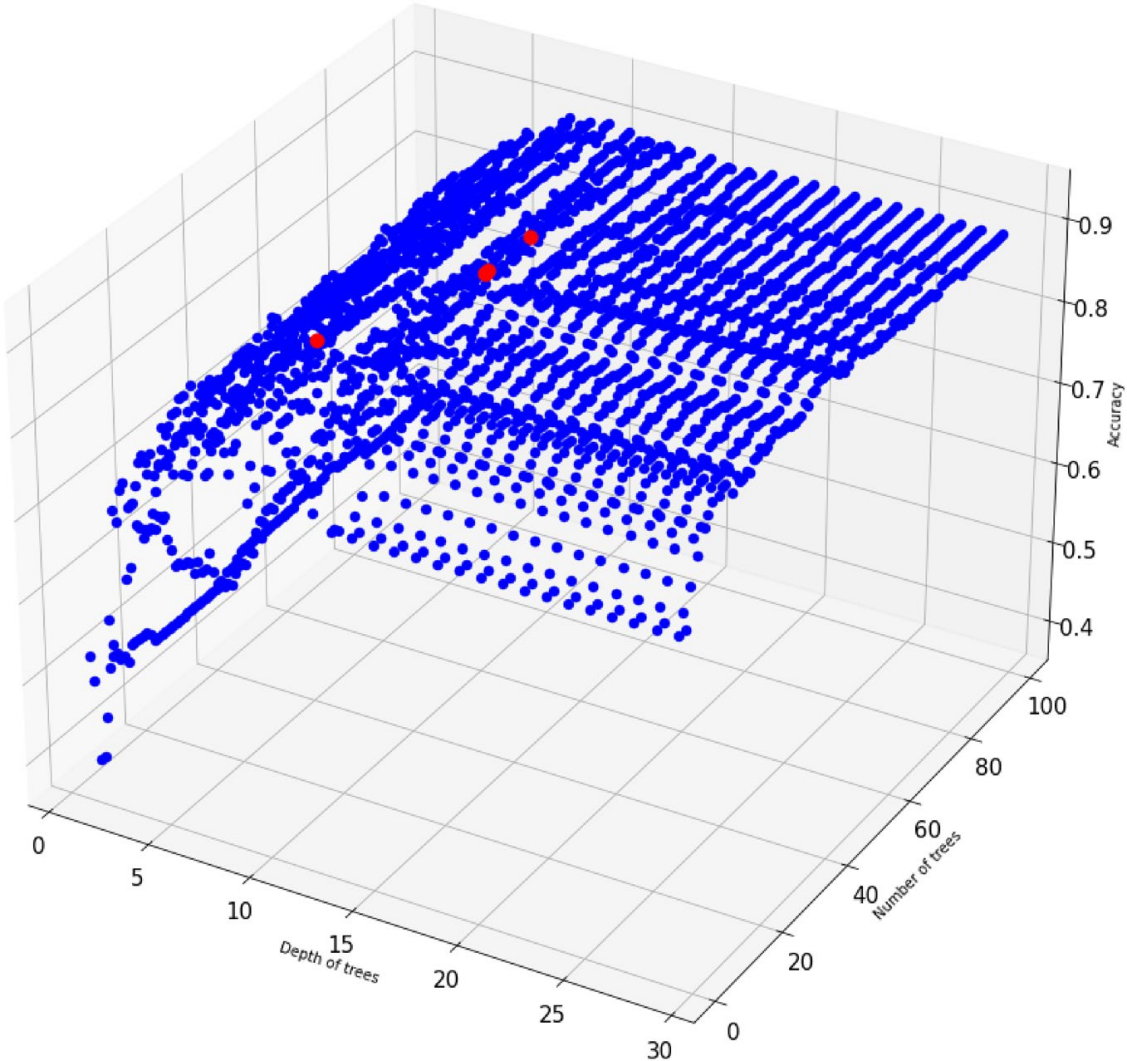
The accuracy scores of random forests with multiple values for its hyper-parameters. Red dots display the maximum accuracy of 92% for random forest models using 15 trees with the maximum depth of 9, or 44 trees with the maximum depth of 12, or 45 trees with the maximum depth of 12, or 57 trees with the maximum depth of 12, or 60 trees with the maximum depth of 12, or 61 trees with the maximum depth of 12, or 63 trees with the maximum depth of 12.

**Figure 6:**
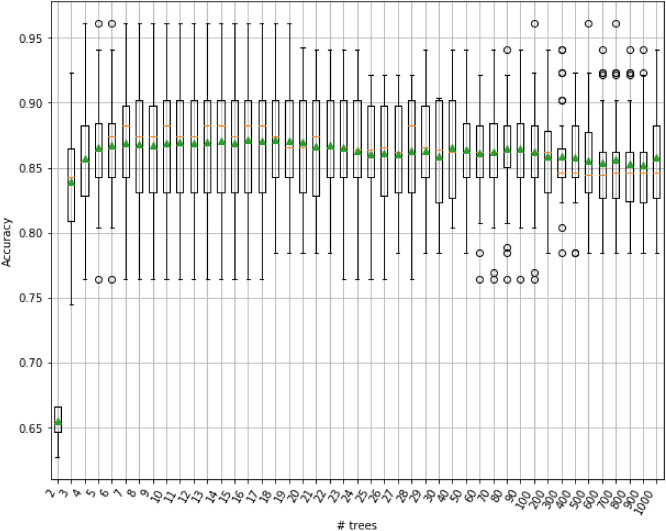
Feature importance scores of Random Forest with 44 trees with a maximum depth of 12.

However, the number of trees and the maximum depth of each tree, hyperparameters, play a very important role in a random forest’s performance. To achieve the best accuracy of the model on the dataset, we tuned the number of trees between 1 and 100 while the maximum depth trees could change between 1 and 30. As the result of hyperparameter-tuning process, the accuracy of a random forest has reached the maximum of 92% and it has been obtained when it contains 15 trees with the maximum depth of 9, or 44 trees with the maximum depth of 12, or 45 trees with the maximum depth of 12, or 57 trees with the maximum depth of 12, or 60 trees with the maximum depth of 12, or 61 trees with the maximum depth of 12, or 63 trees with the maximum depth of 12. Figure 5 displays the accuracy scores of random forests with multiple values for its hyper-parameters. Figure 6 displays feature scores of the random forest model that utilizes 44 trees with a maximum depth of 12.

**Figure 7:**
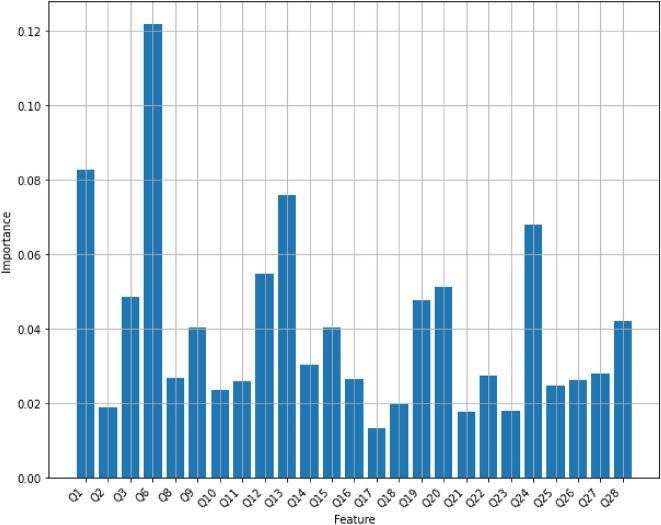
Box plot for the mean accuracy of Gradient Tree Boosting with different number of trees.

**Figure 8:**
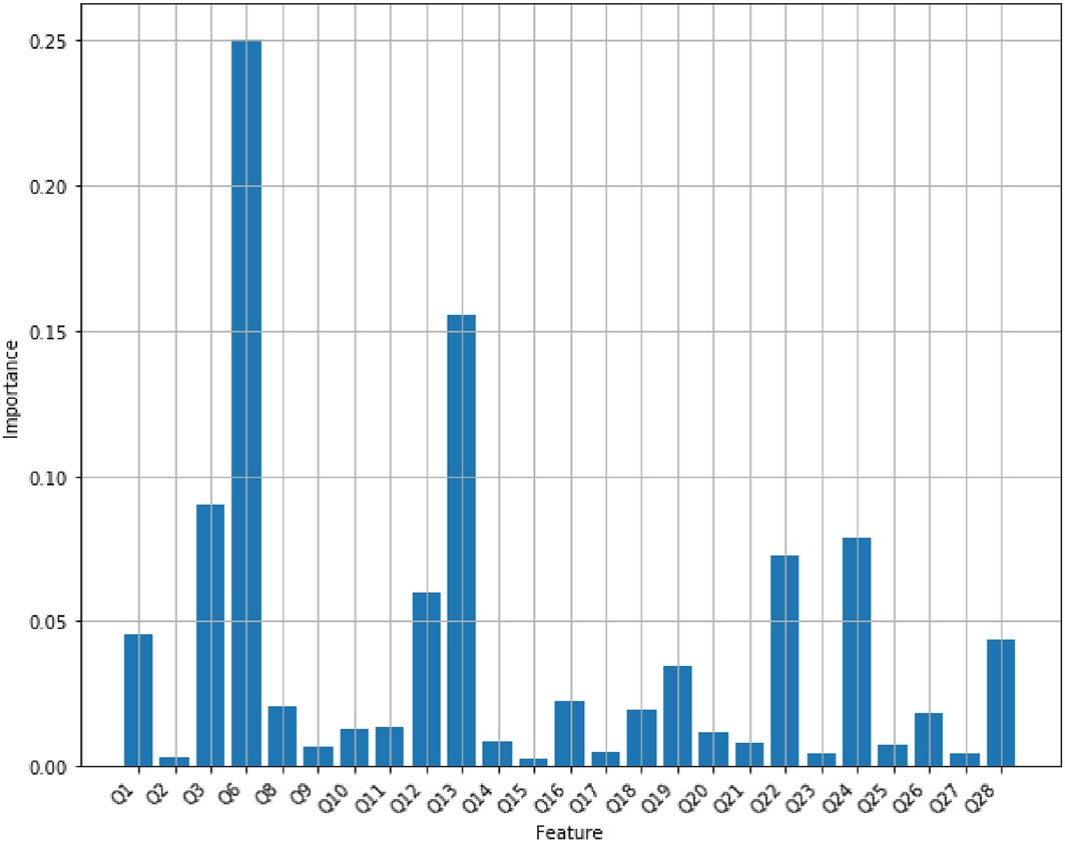
The feature scores of Gradient Tree Boosting with 19 trees.

*Gradient tree boosting* Gradient tree boosting is an ensemble model, which is constructed from many individual decision tree models. Decision trees are added one at a time to the model and fit to minimize the error made by previous trees. In this model, gradient descent or stochastic gradient descent methods are used to minimize the differentiable loss functions. Gradient boosting is one of the most widely used machine learning algorithm on tabular datasets. The number of decision trees is one of the most important hyper-parameters for the Gradient Boosting ensemble algorithm. The depth of the trees and the number of trees can be used efficiently in the ensemble to improve the performance of a model. Figure 7 displays the effect of the number of trees on accuracy by means of a box and whisker plot. It turns out that if we change the number of trees between 16 and 19, the mean accuracy scores of ensemble models do not change significantly. The mean accuracy of the ensemble model on the Mental Health Data meets its maximum 87.1% when the number of trees is between 16 and 19. Figure 8 displays the feature scores Gradient Tree Boosting with 19 trees.

Gradient boosted trees have become the most widely used machine learning algorithms when it comes to tabular data. There are several popular boosting algorithms such as XGBoost, CatBoost and LightGBM. However, XGBoost, CatBoost and LightGBM algorithms differ from one another in the implementation of the boosted trees algorithm and the splitting mechanism.

**Figure 9:**
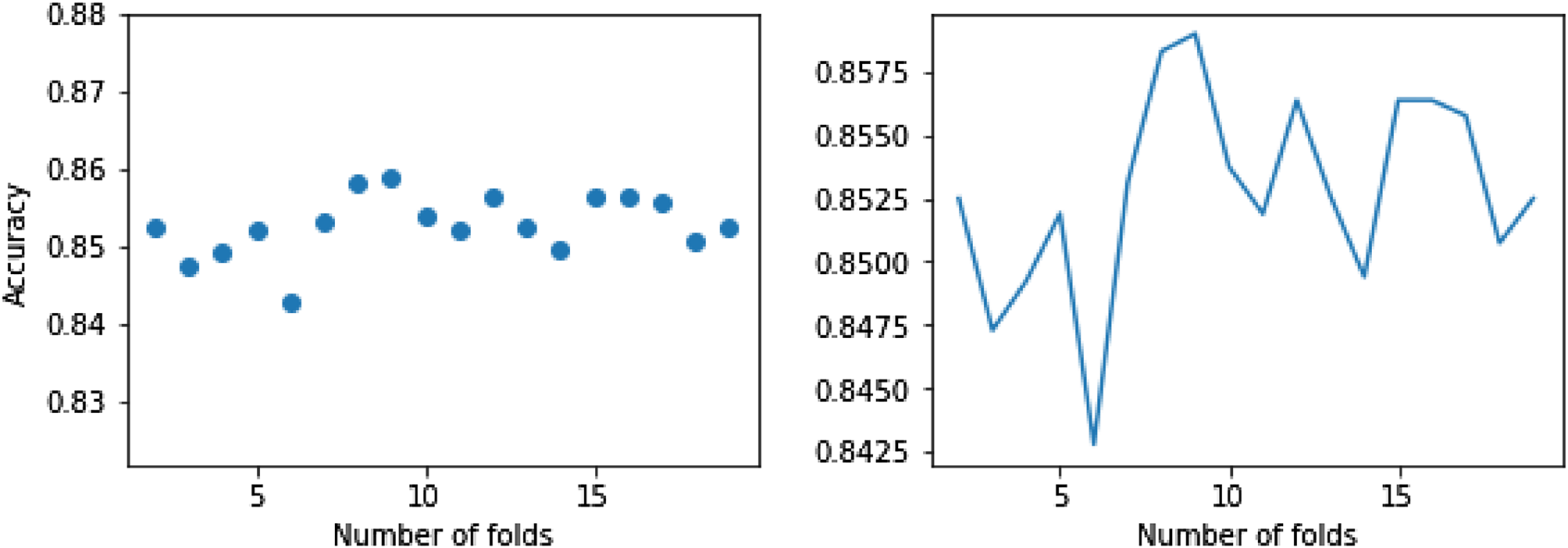
The mean accuracy of XGBoost using k-fold cross-validation.

**Figure 10:**
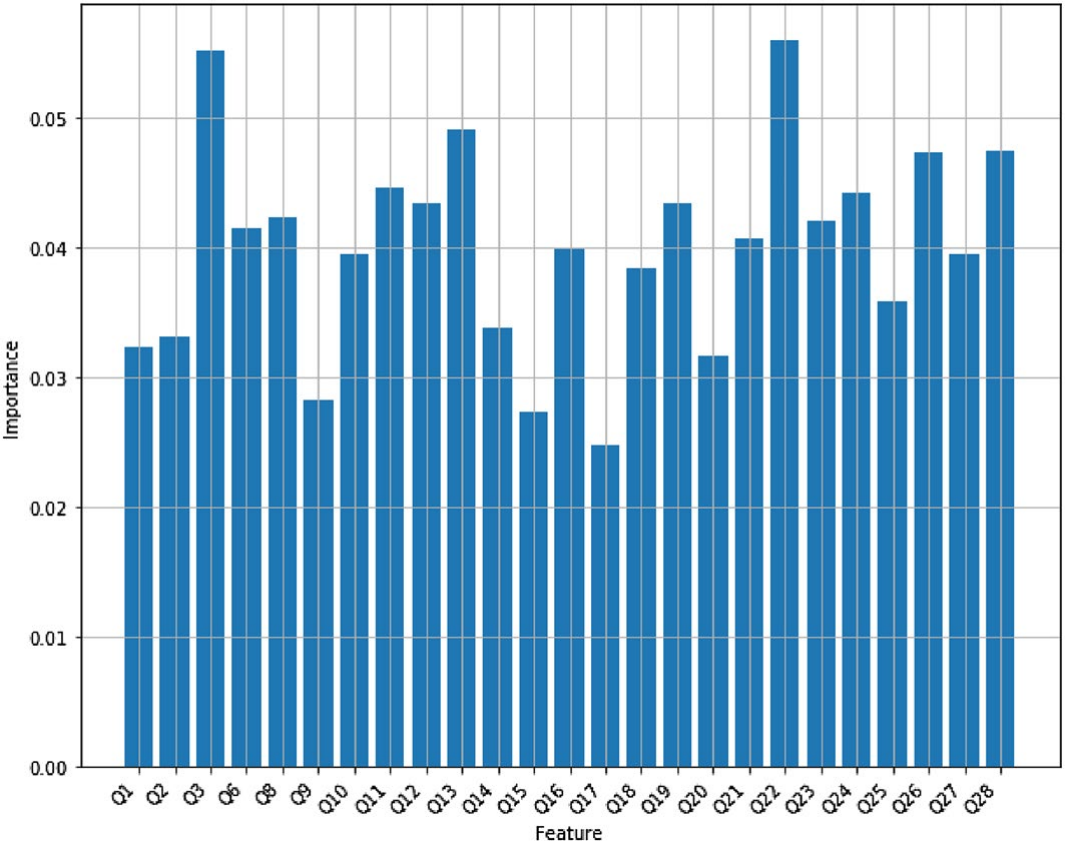
The feature scores of XGBoost using $k=9$ cross-validation.

*eXtreme gradient boosting (XGBoost)* XGBoost, introduced by Tianqi Chen^27,28^, is a widely used machine learning algorithm that implements machine learning algorithms using the Gradient Boosting framework with high accuracy and speed. It was initially introduced to improve GBM’s training time. It provides a parallel tree boosting framework to solve so many multiclass classification problems. Cross-validation is a popular method to find a better accuracy for a model rather than a simple train/test split. The cross-validation procedure is shuffling the data set randomly, splitting the data set in *k* groups, taking each group as a test data and feeding the rest to learn a model and find its accuracy, and then calculating and returning the mean of all *k* obtained accuracy. If the value for *k* is assigned to be the number of observations, then it is called leave-one-out cross-validation. Figure 9 displays the accuracy scores of XGBoost with multiple folds ($2\le k\le 20$) for cross-validation on the Mental Health Data. It turns out that XGBoost with k=9 folds gives the best accuracy, approximately 86%. Figure 10 displays the feature scores of the XGBoost model using $k=9$ cross-validation on the Mental Health Data.

**Figure 11:**
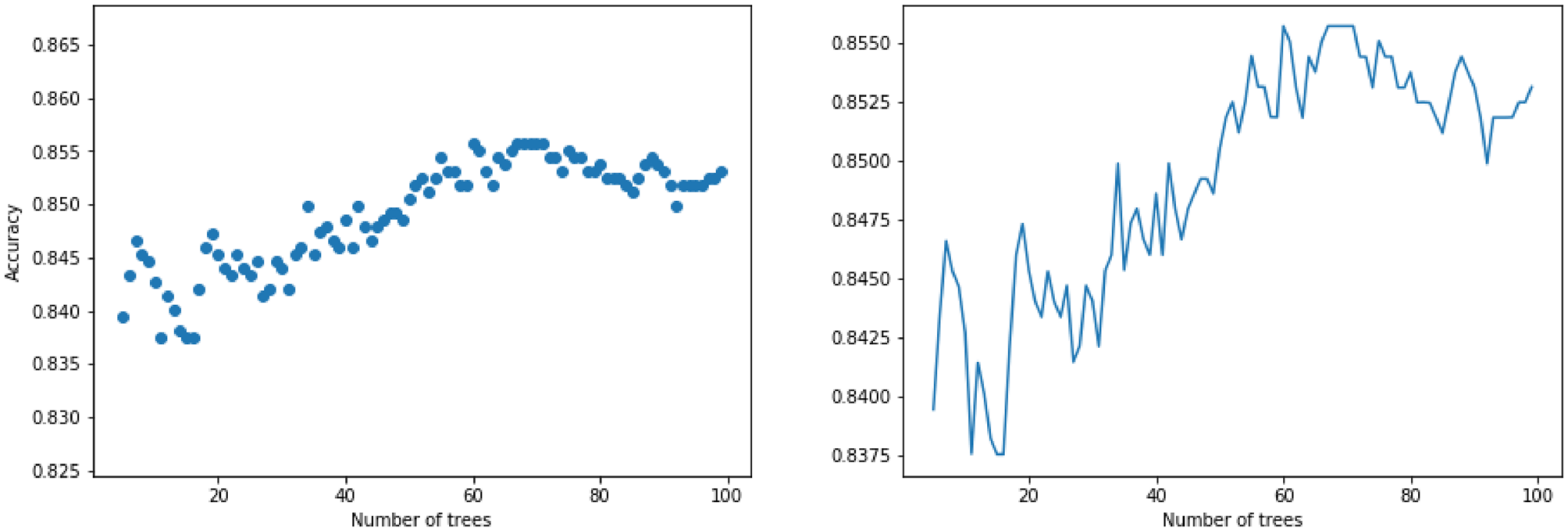
The mean accuracy of CatBoost using several trees.

**Figure 12:**
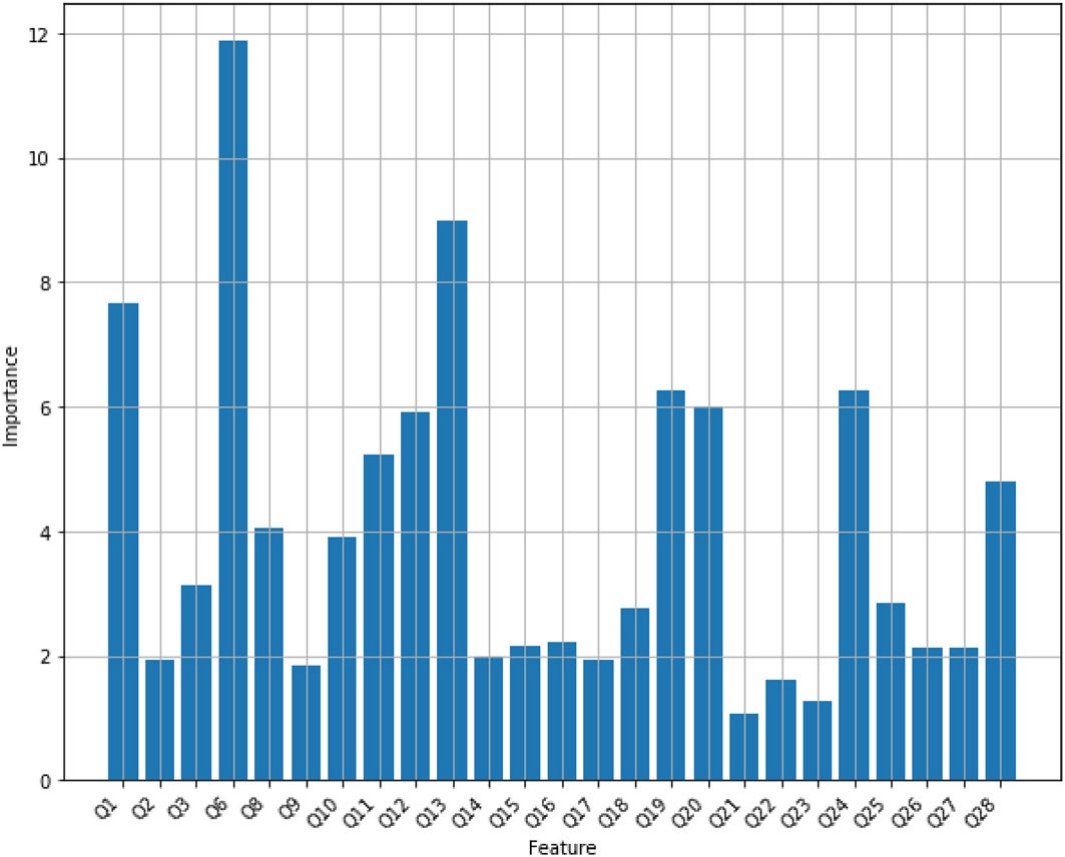
The feature scores of the CatBoost model using 60 trees.

*CatBoost* CatBoost^29^ is another algorithm for gradient boosting on decision trees. CatBoost trains trees sequentially, so that each successive tree is built with reduced loss compared to the previous trees. CatBoost can be used directly on categorical variables without any explicit pre-processing to convert categories into numbers. The number of trees is one of the most important hyper-parameters in CatBoost algorithm. Figure 11 displays the accuracy of CatBoost model on the Mental Health dataset for multiple number of trees. It turns out that CatBoost with 60 trees gives the best accuracy, approximately 85.50%. Figure 12 displays the feature scores of the CatBoost model using 60 trees.

*Light gradient boosted machine (LightGBM)* LightGBM, introduced by Guolin Ke et al.^30^ in collaboration with Microsoft, is another strong learner that applies the boosting framework, which train trees sequentially instead of simultaneously, like CatBoost. LightGBM uses gradient-based one-side sampling (GOSS) that aims to find a good balance between increasing speed by reducing the number of data points and holding the accuracy for learned trees. LightGBM differs from XGBoost and CatBoost in the way that it works based on gradient-based one-side sampling (GOSS) and Exclusive Feature Bundling (EFB). GOSS excludes a significant proportion of data instances with small gradients, using the rest to estimate the information gain in individual trees. Guolin Ke et al.^30^ prove the data instances with larger gradients play a more important role in the computation of information gain, and thus GOSS can obtain an accurate estimation of the information gain with a much smaller data size. EFB bundles mutually exclusive features to reduce the number of features. Mutually exclusive features are features that rarely take nonzero values simultaneously, such as one-hot encoded features. Guolin Ke et al.^30^ prove that finding the optimal bundling of exclusive features is NP-hard, but a greedy algorithm can achieve a good approximation ratio, effectively reducing the number of features without hurting accuracy of the model.

**Figure 13:**
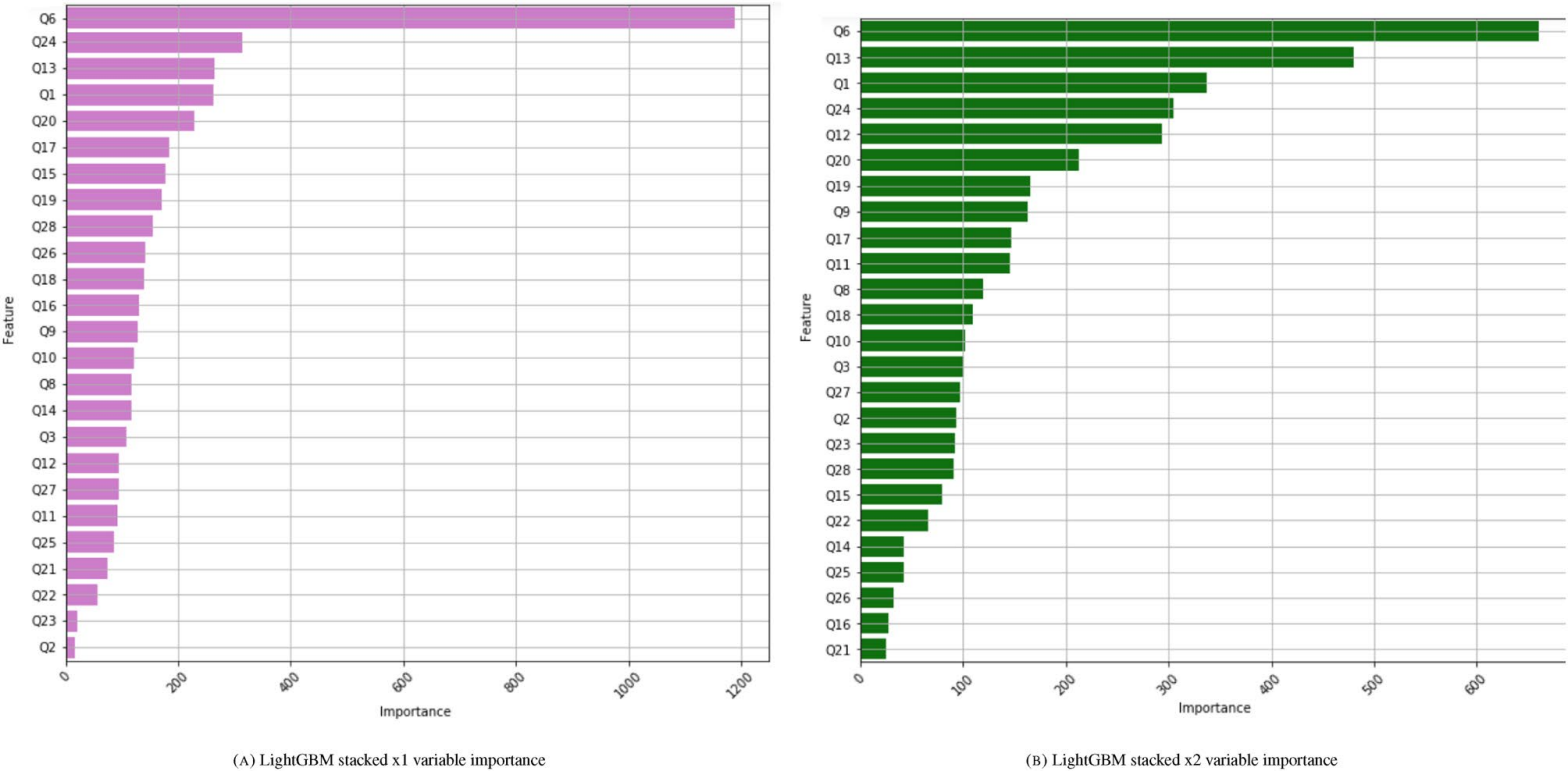
LightGBM.

Tables 3 and 4 display the accuracy scores of LightGBM model stacked once and twice, respectively, on the Mental Health Data. The model uses 200 gradient-boosted decision trees (GBDT) all limited to a maximum depth of 8. Figure 13 displays the feature scores of LightGBM model stacked with one and two variables on the Mental Health data. The first four important features for LightGBM stacked with one and two variables are shown in Table 5.

**Table 3:** Stacked LightGBM Scores.

	Precision	Recall	f1-score	Support
Accuracy			0.81	154
Macro avg	0.46	0.44	0.43	154
Weighted avg	0.77	0.81	0.78	154

**Table 4:** 2x Stacked LightGBM scores.

	Precision	Recall	f1-score	Support
Accuracy			0.91	180
Macro avg	0.46	0.46	0.46	180
Weighted avg	0.90	0.91	0.90	180

**Table 5:** The most important variables for LightGBM.

LightGBM stacked with 1 variables	
Rank	Feature
#1	Q6: What is your role in the healthcare field? (e.g. psychologist, physician, nurse)
#2	Q13: Approximately how many hours did you sleep on an average work night in the last week?
#3	Q20: In the last month, approximately how often did you have a drink containing alcohol?
#4	Q24: How many hours of COVID-19 related news or social media are you consuming on average per day?

LightGBM stacked with 2 variables	
Rank	Feature
#1	Q6: What is your role in the healthcare field? (e.g. psychologist, physician, nurse)
#2	Q13: Approximately how many hours did you sleep on an average work night in the last week?
#3	Q1: What is your age?
#4	Q24: How many hours of COVID-19 related news or social media are you consuming on average per day?

**Figure 14:**
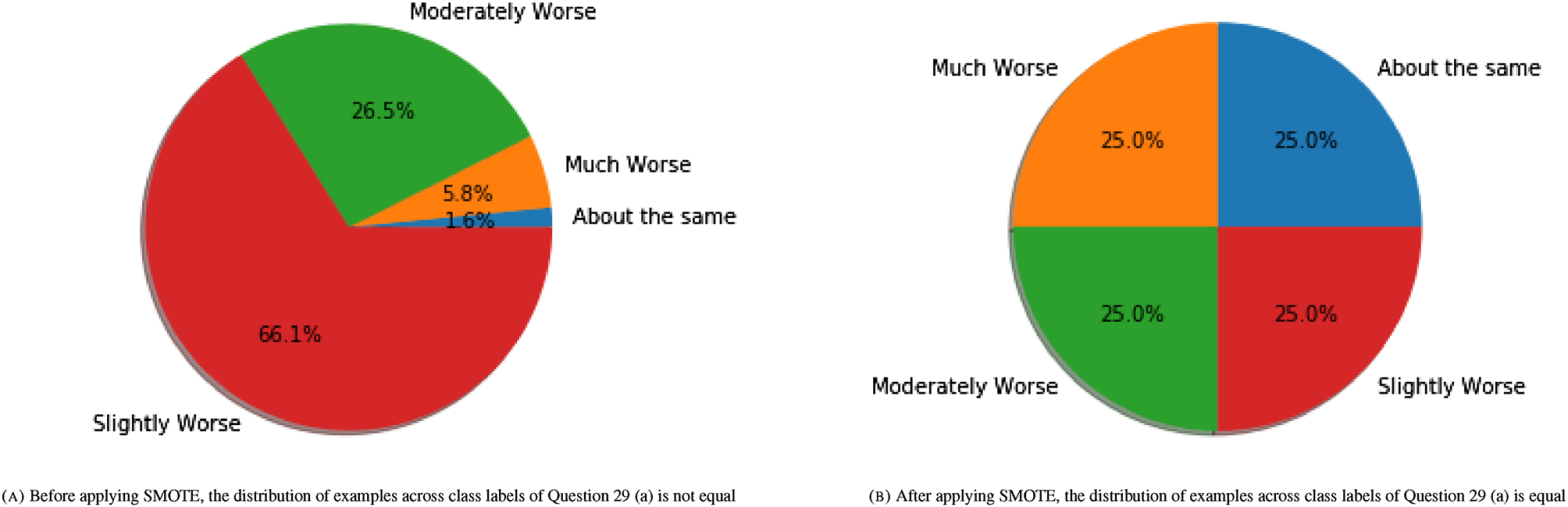
Applying SMOTE to oversample all classes to the number of examples in the majority class.

**Figure 15:**
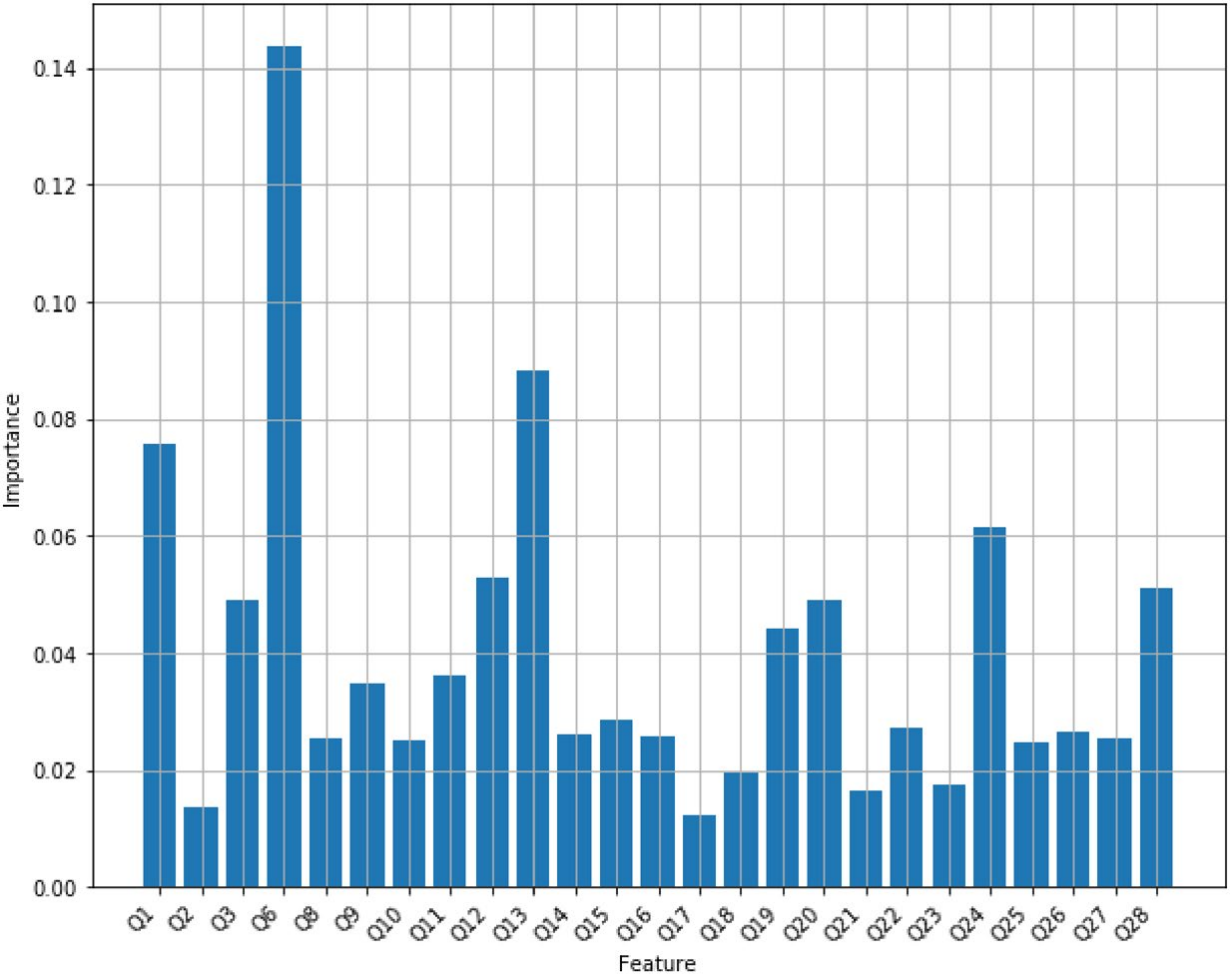
Feature importance scores of SMOTE Random Forest.

**Figure 16:**
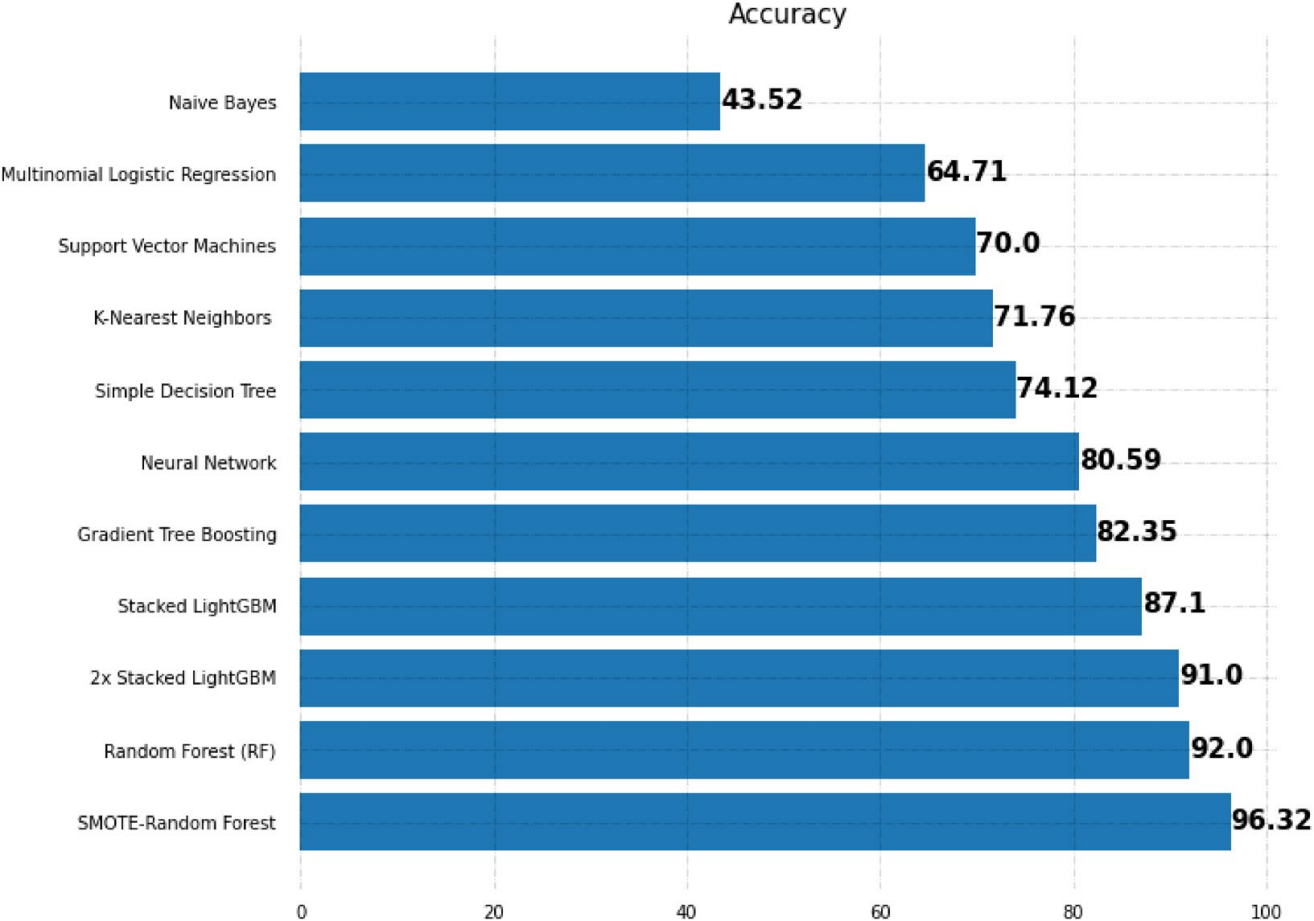
Improving the Randome Forest accuracy by means of SMOTE.

*Synthetic minority oversampling technique* Note that the categories of the target variable, Question 29 (a), are not approximately equally represented. Nitesh Chawla et al.^31^ proposed a technique called the Synthetic Minority Oversampling Technique, or SMOTE, for synthesizing new examples of the minority classes. The SMOTE is a combination of over-sampling the minority classes and under-sampling the majority classes to achieve a better classifier performance. Since there are few examples with labels belong to some classes, we remove them before we apply SMOTE to avoid misleading the model (see Fig. 14A). Applying the SMOTE (see Fig. 14B) on the Mental Health data to oversample all classes to the number of examples in the majority class on Random Forest improves accuracy of the model to 96.32% (see Fig. 16) with questions 6, 13, 1 and 24 as the most important features, respectively (see Fig. 15). The first four important features for the SMOTE Random Forest are shown in Table 6.

**Table 6:** The first four most important features for the regular SMOTE Random Forest.

SMOTE random forest	
Rank	Feature
#1	Q6: What is your role in the healthcare field? (e.g. psychologist, physician, nurse)
#2	Q13: Approximately how many hours did you sleep on an average work night in the last week?
#3	Q1: What is your age?
#4	Q24: How many hours of COVID-19 related news or social media are you consuming on average per day?

**Figure 17:**
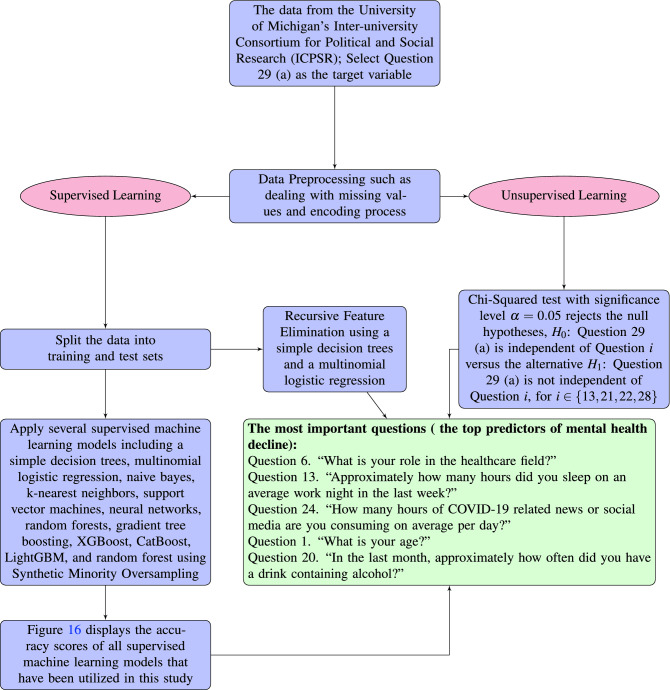
Multiple statistical and machine learning models and techniques such as Decision Trees, Multinomial Logistic Regression, Naive Bayes, k-Nearest Neighbors, Support Vector Machines, Neural Networks, Random Forests, Gradient Tree Boosting, XGBoost, CatBoost, LightGBM, Synthetic Minority Oversampling, and a Chi Squared Test have been used to identify the most important factor in predicting the mental health decline of a frontline worker. It turns out that the top predictors are the healthcare role the individual is in (Nurse, Emergency Room Staff, Surgeon, etc.), followed by the amount of sleep the individual has had in the last week, the amount of COVID-19 related news an individual has consumed on average in a day, the age of the worker, and the usage of alcohol and cannabis.

Figure 16 displays the accuracy scores of all supervised machine learning models that applied on the Mental Health data in this section. Top predictors for mental health analysis have been identified from different approaches. Among all the approaches, the random forest using SMOTE is the model that has identified the maximum top predictors. Figure 17 summarizes the methodology and the results in this section.

## Discussion

Upon the examination of the top predictors from the many different models, we have identified four main questions as well as a group of questions (questions 18–22) as highly predictive of mental health decline in frontline workers. With the emergence of new strains of COVID-19 such as the Delta and Omicron variants, we may be able to learn from our past and improve the working environment for frontline workers such that their mental health does not decline much, if at all.

Our analysis concludes that Question 6: “What is your role in the healthcare field?” is the most important predictor of mental health decline. Both stacked LightGBM models, the Random Forest with and without SMOTE, CatBoost, and Gradient Tree Boosting all conclude that this is the most important predictor. This result is unsurprising, as we expect a frontline worker dealing with extremely sick COVID-19 patients would suffer a greater mental health decline than a child pediatrician who is not interacting with COVID-19 patients. During the COVID-19 pandemic, frontline workers’ roles have been expanded dramatically.

We also find Question 13: “Approximately how many hours did you sleep on an average work night in the last week?”as the second most important predictor. The Random Forest with and without SMOTE, Gradient Tree Boosting, CatBoost, both LightGBM models, and the Chi Squared analysis find this is a highly important predictor. This analysis reveals that COVID-19 can have large, potentially deadly, effects on frontline workers even without contracting the virus. One of the “hidden”effects of COVID-19 on frontline workers is the sleep pattern and sleep quality changes since the stress of their jobs takes a toll on their body both mentally and physically. Moreover, during the COVID-19 pandemic, due to a lack of frontline workers or an excess of patients sick with the virus, the frontline workers have been asked to work disproportionately longer shifts. Frontline workers’ performance and level of care may increase if they resume their normal sleep schedules as well as the mental health of their workers improving during a pandemic.

Our analysis finds that Question 24: “How many hours of COVID-19 related news or social media are you consuming on average per day?” is a highly important predictor of mental health decline in frontline workers. The Random Forest with and without SMOTE, Gradient Tree Boosting, both LightGBM models, and the Recursive Feature Elimination utilizing a decision tree all find this is in the top 5 most important predictors. During the pandemic, there is seldom positive news regarding the virus circulating on the on social media, with many headlines pointing to new variants that are more contagious or deadly than the one before it. It is not surprising to see that consuming more and more of this media can lead a front-line worker to doubt that we would ever beat COVID-19, which causes a decrease in mental health. One could purposefully try to limit their consumption of such media and may find that their mental health and possibly even physical health (such as sleep patterns) may improve.

Looking at the top predictors of the Random Forest with and without SMOTE, CatBoost, and both LightGBM models, we find that Question 1: “What is your age?” is one of the top five most important predictors of mental health decline in frontline workers. We hypothesize that workers that are younger and less familiar with intense medical situations may be more prone to experiencing mental health decline upon encountering COVID-related stressful work environment as well as immense and unprecedented pressures. Healthcare institutions should encourage their less and more experienced staff to work together, allowing for the younger staff to learn helpful tips for their job as well as enabling them to reach out to the knowledgeable staff if they feel as though they are beginning to have mental health issues.

Lastly, we find that a group of questions regarding alcohol and cannabis use stand out as highly predictive in many different models such as the Recursive Feature Elimination with both Decision Trees and Multinomial Regression, the Chi Squared test, the Random Forest with and without SMOTE, XGBoost, CatBoost, and both LightGBM models. This result is intuitive because many people may turn to different methods of coping with the extreme stress that they feel during their day-to-day lives combating COVID-19 and its many variants. Healthcare institutions may find that offering counseling services and other stress relief programs may decrease the usage of Alcohol and Cannabis in their frontline workers, improving both their mental and physical health.

Our analysis has demonstrated that COVID-19 has taken a toll on the mental and physical health of frontline workers. With the emergence of many variants and subvariants, large healthcare institutions can learn from the past to improve conditions and offer their frontline workers a better chance of improving/maintaining their mental and physical health. We know some of the physical symptoms that come from contracting COVID-19, but what we have found is that there exist some “hidden” effects of the virus that do not necessarily come from direct contact. These “hidden” effects may be partially preventable, and in the face of new variants that could cause a resurgence of the virus, healthcare institutions must take steps to prevent these “hidden” symptoms from becoming problems for our frontline workers.

## Conclusion and future work

We have presented an analysis of a COVID-19 mental health survey data obtained from the University of Michigan Inter-University Consortium for Political and Social Research. As part of the analysis, we have utilized a variety of statistical and machine learning models and techniques such as Decision Trees, Multinomial Logistic Regression, Naive Bayes, k-Nearest Neighbors, Support Vector Machines, Neural Networks, Random Forests, Gradient Tree Boosting, XGBoost, CatBoost, LightGBM, Synthetic Minority Oversampling, and a Chi Squared Test. Through the interpretation of the many models applied to the mental health survey data, we have concluded that the most important factor in predicting the mental health decline of a frontline worker is the healthcare role the individual is in (Nurse, Emergency Room Staff, Surgeon, etc.), followed by the amount of sleep the individual has had in the last week, the amount of COVID-19 related news an individual has consumed on average in a day, the age of the worker, and the usage of alcohol and cannabis. Considering the recent identification of the Omicron and Delta variants of COVID-19, we hope that these findings can be utilized by healthcare facilities to help preserve or improve their employee’s mental health.

In future work we would like to aggregate more data on frontline workers, their habits, and their mental health. With more data from a diverse range of locations, we would have the ability to apply even more complex and accurate models to the data, while simultaneously allowing us to make even stronger conclusions about the impacts COVID-19 has on the mental health of frontline workers. Additionally, in future work, we may be able to utilize permutation importance along with the feature importance to shed even more light on the inner workings of the matching learning models. Finally, we would like to identify specific features then utilize generalized linear models to more exactly quantify the relationship the variables have with mental health decline.

We are also interested in analyzing accuracy, speed and feature scores of some machine learning models on the COVID-19 mental health data when we replace the most common optimizers such as GD, SGD or Limited-memory BFGS with the ones that are introduced recently^32,33^.

## Supplementary Information

Supplementary Information.

## Data Availability

The data that support the findings of this study are available from the University of Michigan’s Inter-University Consortium for Political and Social Research, which is collected by Deirdre Conroy^14,15^, at https://www.openicpsr.org/openicpsr/project/127081/version/V1/view?path=/openicpsr/127081/fcr:versions/V1&type=project^16^.
