# Supplementary material for: A machine learning analysis of COVID-19 mental health data
Source: Sci Rep. 2022 Sep 2;12:14965. doi: 10.1038/s41598-022-19314-1 (PMC9438361; doi:10.1038/s41598-022-19314-1)
Supplement: Supplementary file 1 — Supplementary Information. [file 41598_2022_19314_MOESM1_ESM.pdf]

## AppendixA. Some important features in the mental health data

| Question | Levels | Question | Levels |
|---|---|---|---|
| Q1: What is your age? | 10-20<br>21-30<br>31-40<br>41-50<br>51-60<br>61-70<br>71-80<br>81-90 | Q6: What is your role in the healthcare field? | Psychologist<br>Physician<br>Nurse<br>Psychiatrist<br>Social Worker<br>Research coordinator/manager/Public health/Staff<br>etc. |
| Q9: Are you conducting clinical video visits with your patients from your home? | No<br>Yes | Q10: Does your work require you to follow fixed work schedule while working from home? | No<br>Yes |
| Q14: Has the number of your worked hours per week changed? | No<br>Yes | Q15: Have you varied your work schedule? | No<br>Yes |
| Q16: Have your sleep patterns changed? | No<br>Yes | Q17: Has the number of naps you are taking changed? | No<br>Yes |
| Q18: Has the amount of alcohol you are consuming changed? | No<br>Yes | Q19: In January 2020, approximately how often did you have a drink containing alcohol? | 4 or more times a week<br>2-4 times a month<br>2-3 times a week<br>Once a month or less<br>Never |
| Q20: In the last month, approximately how often did you have a drink containing alcohol? | 4 or more times a week<br>2-4 times a month<br>2-3 times a week<br>Once a month or less<br>Never | Q21: In January 2020, approximately how often did you use marijuana/cannabis? | 4 or more times a week<br>2-4 times a month<br>2-3 times a week<br>Once a month or less<br>Never |

| Question | Levels | Question | Levels |
|---|---|---|---|
| Q22: In the last month, approximately how often did you use marijuana/cannabis? | 4 or more times a week<br>2-4 times a month<br>2-3 times a week<br>Once a month or less<br>Never | Q23: Has the amount of news you are consuming increased since the end of Feb, 2020? | No<br>Yes |
| Q24: How many hours of COVID-19 related news or social media are you consuming on average per day? | 4 or more times a week<br>2-4 times a month<br>2-3 times a week<br>Once a month or less<br>Never | Q25: Have you had more "screen time" e.g. use of smartphone, tablet, etc. around bedtime? | No<br>Yes |
| Q26: Have you changed your movement/exercise? | No<br>Yes | Q27: Has the quality of your diet changed? | No<br>Yes |
| Q28: Has the amount of food you have been eating per day changed? | No<br>Yes | Q29: Has your mood changed? | No<br>Yes |
| Q8: Are you currently conducting your job mostly from home now? | No<br>Yes | Q29 (a): Please tell us how your mood has changed. My mood has been: | Much better<br>Moderately better<br>Slightly better<br>About the same<br>Slightly worse<br>Moderately worse<br>Much worse |

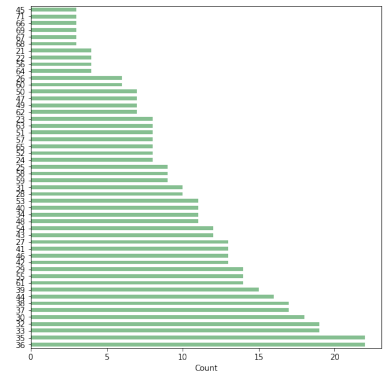


(A) Q1: What is your age?

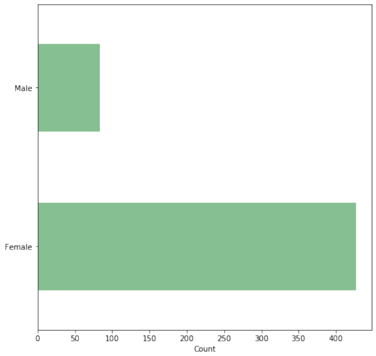


(B) Q2: What is your gender?

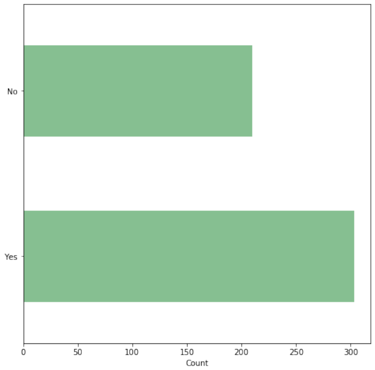


(C) Q8: Are you currently conducting your job mostly from home now?

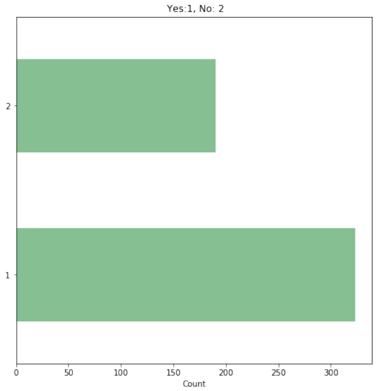


(D) Q9: Are you conducting clinical video visits with your patients from your home?

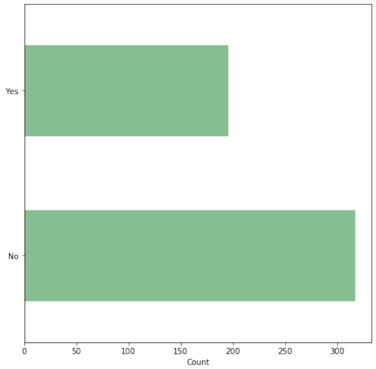


(E) Q10: Does your work require you to follow a fixed work schedule while working from home?

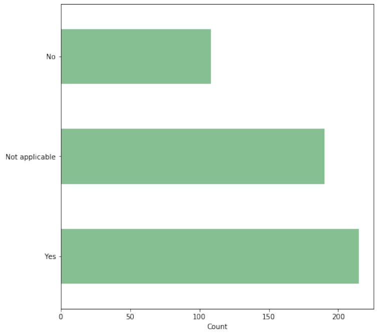


(F) Q11: Are children home from school in the house?

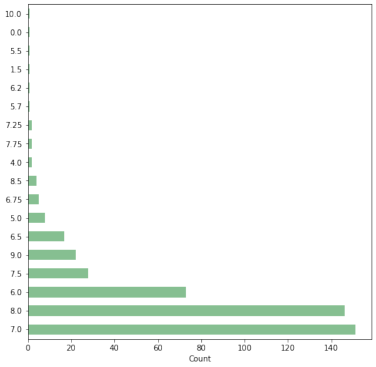


(G) Q12: Approximately how many hours did you sleep on an average work night in January 2020?

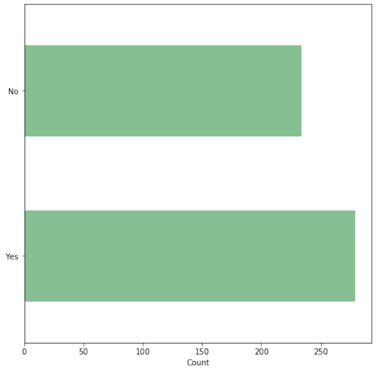


(H) Q14: Has the number of your work hours per week changed?

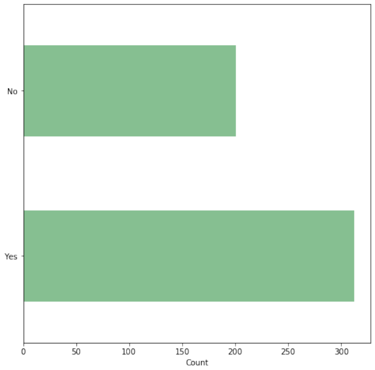


(I) Q15: Have you varied your work schedule?

FIGURE A.1 Frequency bar charts after the preprocessing phase

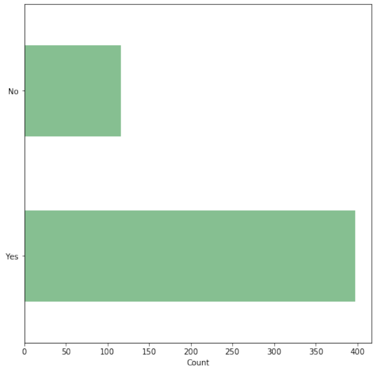


(A) Q16: Have your sleep patterns changed?

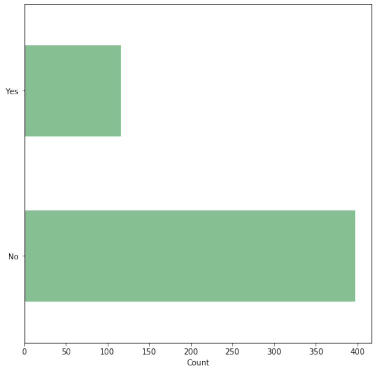


(B) Q17: Has the number of naps you are taking changed?

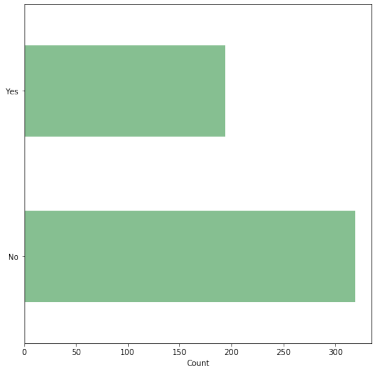


(C) Q18: Has the amount of alcohol you are consuming changed?

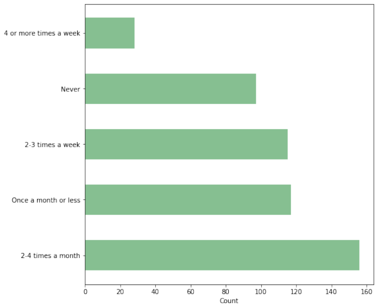


(D) Q19: In January 2020, approximately how often did you have a drink containing alcohol?

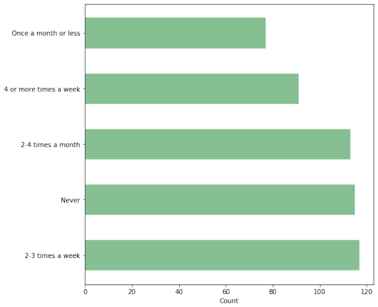


(E) Q20: In the last month, approximately how often did you have a drink containing alcohol?

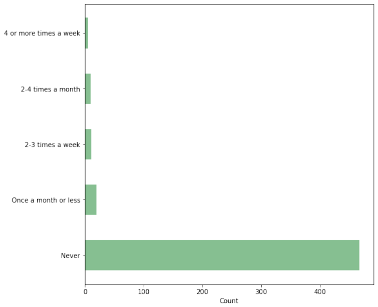


(F) Q21: In January 2020, approximately how often did you use marijuana/cannabis (recreational or medical)?

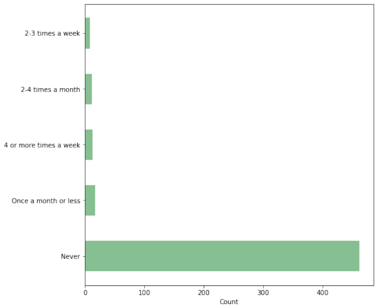


(G) Q22: In the last month, approximately how often did you use marijuana/cannabis (recreational or medical)?

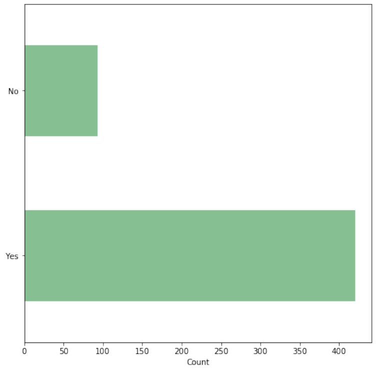


(H) Q23: Has the amount of news you are consuming increased since the end of Feb, 2020?

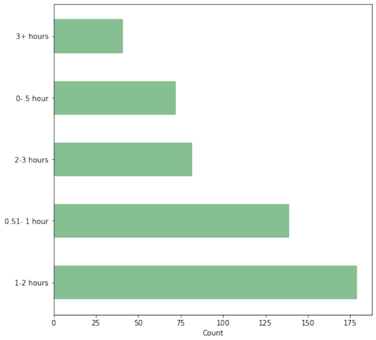


(I) Q24: How many hours of COVID-19 related news or social media are you consuming on average per day?

FIGURE A.2 Frequency bar charts after the preprocessing phase

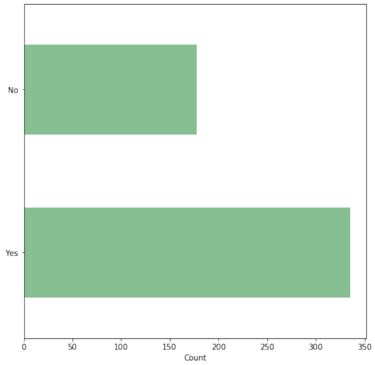


(A) Q25: Have you had more "screen time" (e.g. use of smartphone, tablet, etc.) around bedtime?

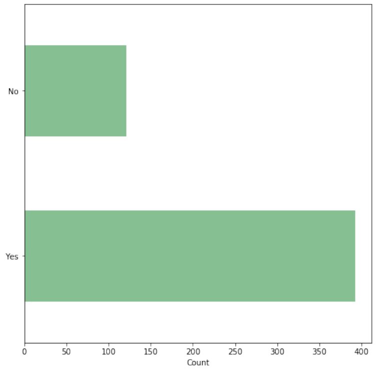


(B) Q26: Have you changed your movement/exercise?

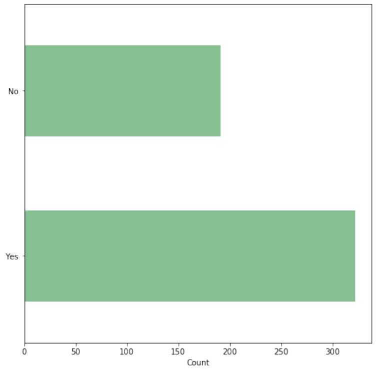


(C) Q27: Has the quality of your diet changed?

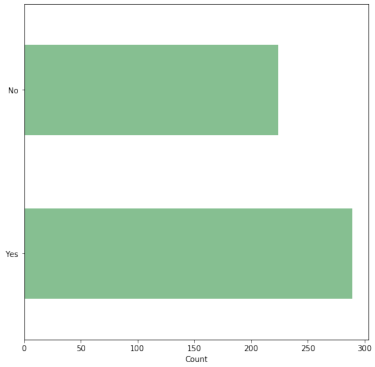


(D) Q28: Has the amount of food you have been eating per day changed?

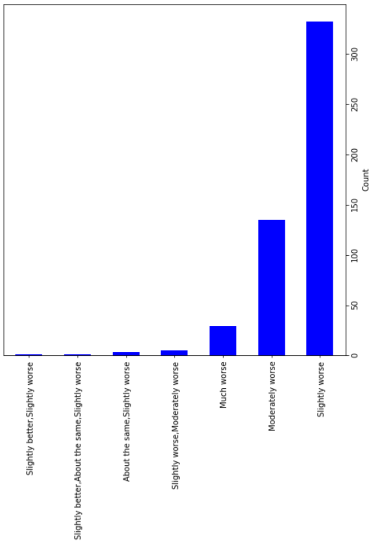


(E) Q29 (a): Please tell us how your mood has changed. My mood has been:

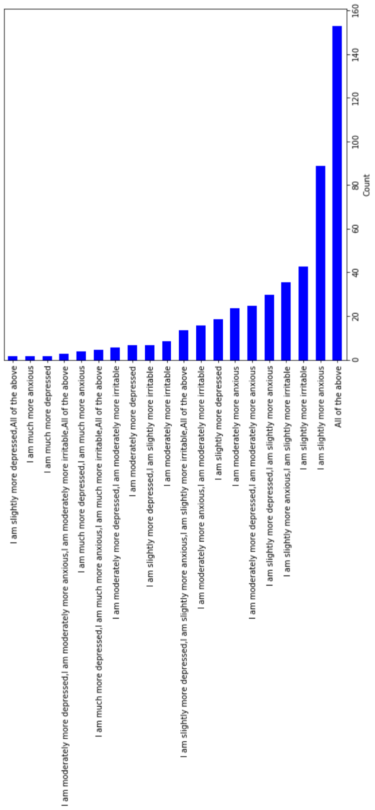


(F) Q29 (b): Please tell us how your mood has worsened

Figure A.3 Frequency bar charts after the preprocessing phase
